# Amastigote Synapse: The Tricks of *Trypanosoma cruzi* Extracellular Amastigotes

**DOI:** 10.3389/fmicb.2018.01341

**Published:** 2018-06-27

**Authors:** Alexis Bonfim-Melo, Eden R. Ferreira, Pilar T. V. Florentino, Renato A. Mortara

**Affiliations:** ^1^Departamento de Microbiologia, Imunologia e Parasitologia, Escola Paulista de Medicina, Universidade Federal de São Paulo, São Paulo, Brazil; ^2^DNA Repair Lab, Biomedical Sciences Institute II, Universidade de São Paulo, São Paulo, Brazil

**Keywords:** *Trypanosoma cruzi*, actin cytoskeleton, microvesicles, host–parasite interactions, Ssp-4 glycoprotein, Rho GTPases, ERM proteins

## Abstract

To complete its life cycle within the mammalian host, *Trypanosoma cruzi*, the agent of Chagas’ disease, must enter cells. Trypomastigotes originating from the insect vector (metacyclic) or from infected cells (bloodstream/tissue culture-derived) are the classical infective forms of the parasite and enter mammalian cells in an actin-independent manner. By contrast, amastigotes originating from the premature rupture of infected cells or transformed from swimming trypomastigotes (designated extracellular amastigotes, EAs) require functional intact microfilaments to invade non-phagocytic host cells. Earlier work disclosed the key features of EA-HeLa cell interplay: actin-rich protrusions called ‘cups’ are formed at EA invasion sites on the host cell membrane that are also enriched in actin-binding proteins, integrins and extracellular matrix elements. In the past decades we described the participation of membrane components and secreted factors from EAs as well as the actin-regulating proteins of host cells involved in what we propose to be a phagocytic-like mechanism of parasite uptake. Thus, regarding this new perspective herein we present previously described EA-induced ‘cups’ as parasitic synapse since they can play a role beyond its architecture function. In this review, we focus on recent findings that shed light on the intricate interaction between extracellular amastigotes and non-phagocytic HeLa cells.

## Cups as Synaptic Junctions and Phagocytosis

**FIGURE 1:**
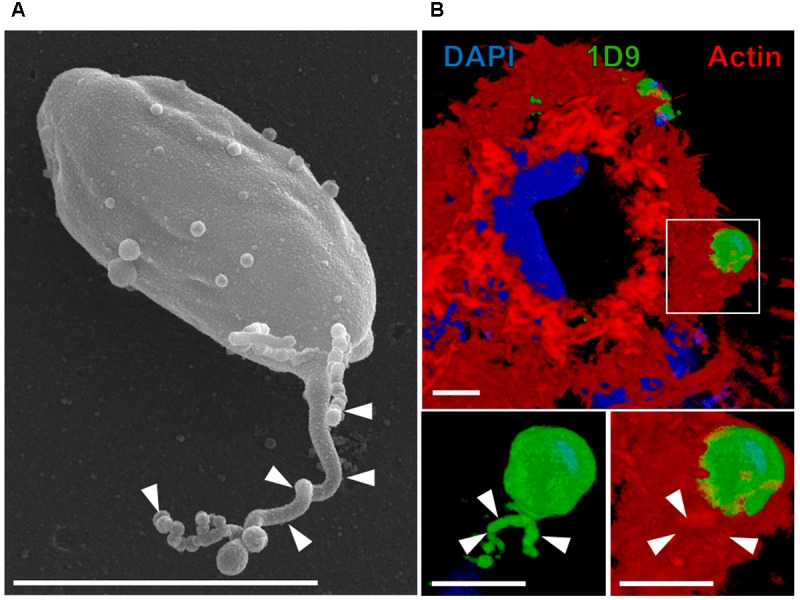
Extracellular amastigotes (EAs) of *T. cruzi* release microvesicles over cellular or non-cellular surfaces. **(A)** EAs can release microvesicles trails (arrowhead) over acellular surfaces such as uncoated transwell membrane (scanning electron microscopy) (Florentino et al., 2018). **(B)** EAs can also secrete microvesicle trails over HeLa cells (Florentino et al., 2018). In this particular interaction, microvesicle trails (arrowheads) were secreted inside the actin-rich cup-like structure induced on the surface of HeLa cells by the EA (3D rendering of confocal microscopy acquisition). Blue: DAPI (4′,6-diamidine-2′-phenylindole dihydrochloride; nuclei and kinetoplasts); green: 1D9 antibody (surface of EAs and microvesicles); red: phalloidin-TRITC (tetramethyl-rhodamine isothiocyanate; actin filaments of HeLa cells) (Florentino et al., 2018). Bars = 3 μm.

**FIGURE 2:**
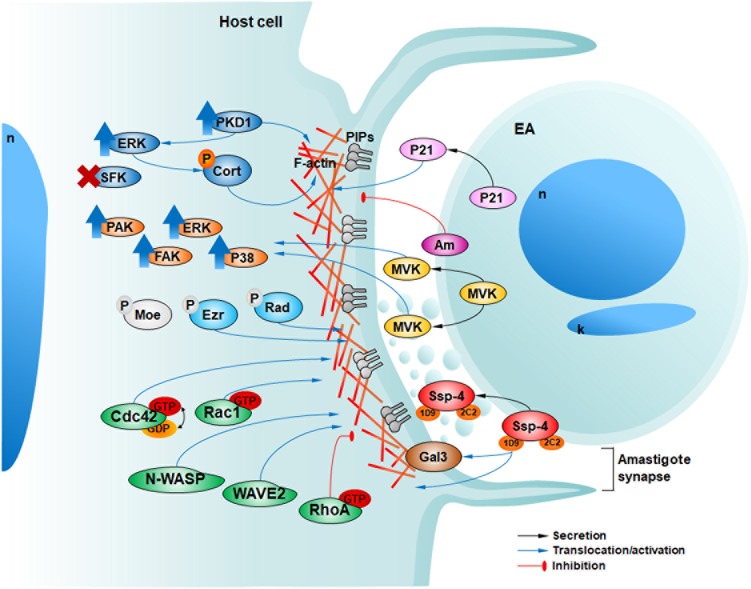
Host and parasite factors recently described by our group involved in HeLa cell invasion by EAs. In 2009, we described the secretion of P21 (pink) and its positive modulation of EA internalization (da Silva et al., 2009). In 2012, we showed that EAs can also engage negative modulators of invasion such as the surface protein amastin (purple, Am) (Cruz et al., 2012). In 2013, we showed that EAs mobilize host phosphoinosites (light and dark gray) along with actin in a phagocytosis-like mechanism (Fernandes et al., 2013). In 2015 Bonfim-Melo et al. (2018) described the activation (upward blue arrows) of PKD1 and ERK but not SFKs upon EA-HeLa interaction with cortactin and PKD1 phosphorylation (orange P) and participation in EA invasion. In 2016 Ferreira et al. (2016) described that TcMVK (yellow) is secreted by EAs promoting their invasion and the activation of diverse actin related host proteins (orange). Additionally, in 2017, we described that ezrin and radixin (cyan), but not moesin (light gray) are involved in actin regulation and EA invasion independent of their phosphorylation (light gray P) activation mechanism (Ferreira et al., 2017). Recently, we observed the participation of Rho GTPases (Rac1 and Cdc42) and their effector proteins regulating actin polymerization signaling and promoting EA invasion, although RhoA inhibited these events (green) (Bonfim-Melo et al., 2018). The surface protein Ssp-4 is differentially expressed among *T. cruzi* strains and can modulate EA invasion through its 1D9 carbohydrate epitope, ligand of host galectin-3 (brown) (Florentino et al., 2018). Finally, EAs can release proteins associated with microvesicles in the confined milieu of the amastigote synapse (Florentino et al., 2018).

When we first described the association of EAs to surface microvilli of HeLa cells (Mortara, 1991), the morphological details of this interaction remained unclear. In 1999, we recognized that during HeLa cell invasion, EAs induce the formation of membranous structures surrounding the parasites that we named ‘cups,’ which later we observed to be suggestive of a phagocytosis internalization process (Procópio et al., 1999; Fernandes et al., 2013). This is the perfect architectural environment for the release of parasite modulating components into a highly confined region being greatly enriched due the small volume milieu followed by the putative clustering of receptor molecules. The cup-like structures triggered by EAs on HeLa cells not only provide a perfect scaffold to drive parasite internalization but also enable the formation of a highly specialized association between the parasite and host cell that resembles an immune synapse (Niedergang et al., 2016). The hypothesis that immune synapses and phagocytosis bear architectural resemblances was recently proposed (Niedergang et al., 2016). Key features shared by immune synapses and phagocytosis, shared by EA-host cell interaction, are actin reorganization (Fritzsche et al., 2017) and microvesicle exchange (Soares et al., 2013). Considering our recent findings on EA biology and host cell signaling we present previously described EA-induced ‘cups’ as parasitic synapse (**Figures 1, 2**) and in the topics below, we discuss molecular mechanisms involved in EA-HeLa cell interactions from both perspectives.

As discussed above, actin recruitment to EA invasion sites is a hallmark identified from very early observations (Mortara, 1991). Actin filaments are the major host component to mediate EA internalization. Cytochalasin D treatment of HeLa or Vero cells strongly inhibits EA internalization (Procópio et al., 1998). Accordingly, the exposure of cytoskeletal elements of HeLa cells infected with EAs early on revealed a meshwork of microfilaments surrounding the parasites (Fernandes et al., 2013). The formation of actin-rich cups surrounding the invading parasites suggested that a phagocytic event may be occurring even in non-phagocytic cell lines. In fact, by mapping phosphatidylinositol lipid chemistry, a hallmark of canonical phagocytosis, our group showed for the first time in 2013 that EAs are strong inducers of phagocytosis in non-phagocytic HeLa cells (Fernandes et al., 2013). Heat-killed EAs or inert particles are poorly internalized by HeLa cells when compared to live EAs (Fernandes et al., 2013) showing that this is a parasite-driven mechanism that leads to specific signaling in host cells (Bonfim-Melo et al., 2015). However, in professional phagocytes such as macrophages, similar internalization is observed among these groups corroborating that EAs rely on actin-dependent phagocytosis-like signaling to invade non-phagocytic cells (Fernandes et al., 2013). As canonical membrane providers in phagocytosis, host vesicular elements also contribute to the early steps of EA invasion, as observed by the recruitment of CD63 (cluster of differentiation 63) and LAMP-1 (lysosomal-associated membrane protein 1) to EA parasitophorous vacuoles and the reduced EA internalization in cells depleted for these proteins (Fernandes et al., 2013). Considering this aspect, EAs differ from trypomastigote (from tissue cultures or metacyclic) forms which invade non-phagocytic cells dependent on host cell lysosome exocytosis and/or PI3k (phosphatidylinositol 3-kinase) activity (de Souza et al., 2010). In the next topics, we discuss the importance of parasite and host components involved in EA internalization by host cells, focusing in recent findings from our research.

## Parasite Ingredients: Amastin, P21, TcMVK, Ssp-4 and Microvesicles

Extracellular amastigotes are *T. cruzi* infective forms that are differentiated in the extracellular milieu. As such, they display features of their flagellated counterparts; that is, they secrete a variety of components that modulate infectivity (Mortara et al., 2005). As indicated previously, EAs must attach to the surface of host cells before invading (Ferreira et al., 2012), and carbohydrate components are the most likely linkers (Barros et al., 1997; da Silva et al., 2006; Florentino et al., 2018). During attachment and the early stages of invasion, parasite components may act as linkers and/or modulators. Among the best-characterized amastigote components are amastin (Cruz et al., 2012), P21 (21 kDa protein) (da Silva et al., 2009) and mevalonate kinase (TcMVK) (Ferreira et al., 2016). Amastin, an amastigote-specific glycoprotein (Teixeira et al., 1994), was unveiled as a negative modulator of EA infectivity, as its expression is higher in the CL strain and lower in the highly infective G strain and *in vitro.* EA invasion in HeLa cells is inhibited by overexpression of EA amastin or after host cell treatment by a soluble recombinant isoform (Cruz et al., 2012). Additionally, amastin accelerates amastigote to trypomastigote transformation during the intracellular cycle. On the other hand, P21 is secreted by all parasite forms, modulates EA invasion, induces host actin filament reorganization and was recently described to hinder angiogenesis in a murine model of chronic Chagas’ disease (da Silva et al., 2009; Teixeira et al., 2017). Evidence suggests that P21 is a general phagocytosis inducer that possibly acts in a PI3k-dependent and ERK (Extracellular signal-regulated kinase)/AKT-independent manner (Rodrigues et al., 2012). Mevalonate kinase is a protein conserved from bacteria to mammals and its primary function relies on the early steps of isoprenoid biosynthesis essential in sterols formation, such cholesterol and ergosterol. In *T. cruzi*, TcMVK is located in glycosomes and in addition to its classical function, TcMVK is secreted by EAs, adheres to host cell membrane and positively modulates their uptake by HeLa cells (Ferreira et al., 2016). Treating mammalian cells with recombinant TcMVK triggers ERK, P38 (p38 mitogen activated protein kinase), FAK (focal adhesion kinase) and PAK (p21-activated kinase), which are important regulators of actin cytoskeleton remodeling although their specific role in host cell actin signaling during EA invasion still remains elusive (Benndorf et al., 1994; Procópio et al., 1998; Vidal et al., 2002; Brunton et al., 2004; Boivin et al., 2012; Navratil et al., 2014; Ferreira et al., 2016).

Ssp-4 (stage-specific surface protein 4) is an 84 kDa GPI (Glycosylphosphatidylinositol)-anchored surface glycoprotein that was originally identified in intra and extracellular amastigotes by Norma Andrews and defined by monoclonal antibody 2C2 (Andrews et al., 1987, 1988). Although 30 years have elapsed, the detailed molecular structure of the Ssp-4 protein core has remained elusive. Recently, we performed proteomic analyses of 2C2 immunoprecipitates and confirmed that the Ssp-4 protein is GPI-anchored and has several *N*- and *O*- glycosylation sites. Interestingly, the infectious EAs of the G strain express lower amounts of Ssp-4 than the less infectious CL strain EAs (at the mRNA and protein levels), and the modulation of EA invasion in HeLa cells by this protein mainly remains in glycosylation residues (Florentino et al., 2018). We showed that the carbohydrate epitope defined by mAb 1D9 binds to HeLa cell galectin-3, which suggested that this may be a putative Ssp-4 receptor (Florentino et al., 2018). Barros et al. (1996) demonstrated that EAs release trails (**Figure 1A**), over modified glass surfaces or host cells, of membranous material covered with Ssp-4. *T. cruzi* metacyclic trypomastigotes and epimastigotes also release microvesicles as an exosome rich shedding process containing a variety of factors involved in metabolism, signaling, nucleic acid binding and parasite survival and virulence (Bayer-Santos et al., 2013) that modulate metacyclic trypomastigote invasion (Clemente et al., 2016). We recently observed that EAs can also secrete these microvesicle trails while coinhabiting with *Leishmania amazonensis* amastigotes in mixed parasitophorous vacuoles (Pessoa et al., 2016) and within the phagocytic cup induced on the HeLa cell surface, possibly modulating parasite invasion (**Figure 1B**) although thus far we cannot directly demonstrate a specific role in EA invasion (Florentino et al., 2018).

## Host Cell Ingredients: Cortactin/PKD1, Rho GTPases/Effector Partners and ERMs

During the interaction and invasion processes, EAs induce a variety of HeLa cell responses that regulate actin polymerization and lead to parasite internalization. One response is the recruitment and phosphorylation of a key component of the actin cytoskeleton, cortactin (Bonfim-Melo et al., 2015). HeLa-EA interaction induces cortactin phosphorylation by ERK but not by SFKs (Src family kinases). ERK-phosphorylated SFK-dephosphorylated cortactin is a well-established cortactin state responsible for actin polymerization via the N-WASP (Neuronal-Wiskott–Aldrich syndrome protein)-Arp2/3 complex pathway which we believe is an effector pathway leading to EA internalization (Martinez-Quiles et al., 2004; Bonfim-Melo et al., 2015). Although SFKs mediate host cell invasion by other parasites and bacteria (Agerer et al., 2003; Chen et al., 2003), we did not observe their activation during invasion by EAs (Bonfim-Melo et al., 2015; Ferreira et al., 2016). This observation contradicts our earlier hypothesis stating that SFKs participate in EA internalization (Ferreira et al., 2012). PKD1 (protein kinase D1) is another host cell protein activated upon EA interaction (Bonfim-Melo et al., 2015). In same study PKD1 phosphorylation of cortactin was not assessed but previous studies (Hausser et al., 2001; Brandlin et al., 2002; Rezaee et al., 2013) corroborate our results showing that PKD1 participates in EA internalization possibly by activating ERK by a still unknown mechanism (Bonfim-Melo et al., 2015) EA internalization in HeLa cells. Notably, using cells overexpressing or depleted for PKD1 or cortactin, respectively, we showed that both molecules promote EA internalization in HeLa cells (Bonfim-Melo et al., 2015).

Other relevant proteins in EA phagocytic cup formation in HeLa cells are Rho GTPases (guanosine triphosphate hydrolases) Rac1 (Ras-related C3 botulinum toxin substrate 1), Cdc42 (cell division control protein 42 homolog) and RhoA (Ras homolog gene family, member A), which are key elements in the reorganization of actin microfilaments. We previously observed that Rac1 is important for the EA invasion of transfected MDCK (Madin-Darby canine kidney) cells (Fernandes and Mortara, 2004). Re-examining the effects of Rho GTPases by depletion (inhibition) or overexpression of constitutively active (CA, enhancer) or dominant negative (DN, inhibitor) forms, we confirmed that among these proteins, Rac1 has a major role in cup formation and parasite invasion in HeLa cells (Bonfim-Melo et al., 2018). Cells overexpressing Rac1-CA had increased EA internalization, whereas a decrease was observed in cells overexpressing Rac1-DN (Bonfim-Melo et al., 2018). For Cdc42, overexpression of WT isoform increased EA internalization while overexpression of CA isoform reduced it (Bonfim-Melo et al., 2018). These observations show that whereas Rac1 activation mediates EA invasion, for Cdc42 cycling between active and inactive states is more important than is its full activation alone, similar to what occurs during canonical phagocytosis in professional phagocytes (Beemiller et al., 2010). To induce actin polymerization, Rac1 and Cdc42 rely on their effector partners, NPFs (nucleating promoting factor) WAVE2 (WASP Family Verprolin-homologous Protein-2) and Neural-Wiskott–Aldrich Syndrome protein (N-WASP), respectively. Depletion of these proteins disturbed actin dynamics and inhibited EA invasion, supporting the hypothesis that Rac1 and Cdc42 pathways, albeit to different extents, modulate cup formation and effective EA invasion (Bonfim-Melo et al., 2018). Coupled participation of Rho GTPases and their effector proteins observed in our studies was also described in macrophage phagocytosis thus corroborating our hypothesis that EAs are strong inducers of phagocytic signaling to invade non-phagocytic cells (Freeman and Grinstein, 2014). These results are also in line with PIP (Phosphatidylinositol phosphate) formation (Fernandes et al., 2013) and cortactin participation described earlier by our group (Bonfim-Melo et al., 2015).

Finally, other components from the host cell that participate in EA invasion are the ERM (ezrin, radixin and moesin) proteins. ERM proteins are linkers of actin filaments to the plasma membrane and are present in diverse actin-rich structures, such as lamellipodia membrane ruffles and microvilli; they are also key elements in cell polarity, motility and cell signaling (Tsukita et al., 1997; Ivetic and Ridley, 2004). ERM proteins can also be found in lipid rafts, specialized domains rich in cholesterol and GPI anchored proteins, known for their participation during EA invasion (Fernandes et al., 2007). Our group demonstrated that ERM proteins are recruited to parasite invasion sites and that depletion of ezrin and radixin inhibits EA invasion. Accordingly, ezrin and radixin overexpression enhances parasite invasion (Ferreira et al., 2017). Despite structural and functional similarity to ezrin and radixin, curiously moesin does not seem to be involved in EA invasion because its depletion or overexpression did not significantly alter parasite invasion in HeLa cells (Ferreira et al., 2017). Our findings also indicated that ERM participation in EA invasion does not depend on its classical threonine C-terminal phosphorylation because overexpression of phospho or dephosphomimetic mutants did not alter the EA uptake when compared to the wild-type isoforms (Hao et al., 2009; Bosk et al., 2011; Ferreira et al., 2017). Consistent with this hypothesis, host cells in contact with EAs do not present increased ERM phosphorylation (Ferreira et al., 2017). Independently of or synergistically to phosphorylation of C-terminal serine, PIP_2_ (phosphatidylinositol 4,5-bisphosphate) binding can induce ERM activation (Hao et al., 2009; Bosk et al., 2011; Ferreira et al., 2017). We propose that this may be the key mechanism driving ERM activation during EA internalization since it corroborates with the sequential formation of membrane phosphoinositides, starting with PIP_2_ (Fernandes et al., 2013). Thus, our results showed that ezrin and radixin promote EA uptake by HeLa cells, independently of their activation by serine phosphorylation mechanism.

## Conclusion

In the present report we discuss recent results related to the mechanisms that lead to EA uptake by host cells since our previous review (Ferreira et al., 2012). We also present actin-rich ‘cups’ as parasitic synapses since EAs can secrete soluble factors and microvesicles inside them and modulate host cell signals controlling actin dynamics. In our current working model of EA invasion into HeLa cells (**Figure 2**), we envisage that parasite components, such as Ssp-4, TcMVK and P21, are released into the parasite synapse as soluble factors or associated with microvesicles and modulate host cell responses, possibly mediated by cell receptors such as galectin-3. EA interaction triggers, in host cells, actin polymerization and remodeling through Rac1 and Cdc42 with the concomitant recruitment of actin microfilaments and diverse actin-binding proteins, such as N-WASP, WAVE2, cortactin and ERMs, to the nascent cup (**Figure 2**).

Despite the novel molecules and signaling cross-talk described in the past years, our group is focused on questions that remain elusive: (i) What are parasite surface molecules responsible for the induction of a phagocytosis-like process leading to EA uptake in non-phagocytic cells? (ii) What are those signaling partners of actin modulating proteins current described in EA invasion, such as Rho GTPases, NPFs, cortactin, and others? (iii) What is the role of the previously described molecules/signaling pathways in EA entry in professional phagocytes?

## Author Contributions

RM conceived the work. AB-M, EF, and PF contributed with experimental data. RM, AB-M, EF, and PF wrote the manuscript.

## Conflict of Interest Statement

The authors declare that the research was conducted in the absence of any commercial or financial relationships that could be construed as a potential conflict of interest.
